# Elucidating osteoporosis response signatures in rheumatoid arthritis using explainable machine learning ensembles

**DOI:** 10.1186/s12891-026-09526-1

**Published:** 2026-01-21

**Authors:** Kaibin Lin, Bing Zhou, Zheng Wang, Yiyue Chen, Shu Li, Zijian Zhou, Fen Li, Qiyuan Luo, Jiafen Liao

**Affiliations:** 1 School of Computer Science, Hunan First Normal University, Changsha, China; 2 Department of Rheumatology and Immunology, The Second Xiangya Hospital of Central South University, Changsha, China; 3 Clinical Medical Research Center for Systemic Autoimmune Diseases in Hunan Province, Changsha, China; 4 Clinical Nursing Teaching and Research Section, The Second Xiangya Hospital of Central South University, Changsha, China

**Keywords:** Rheumatoid arthritis, Osteoporosis, CNN-SVM, SHAP

## Abstract

**Background and objectives:**

Osteoporosis (OP) presents a significant health issue in rheumatoid arthritis (RA) patients, yet existing machine learning (ML) studies on OP prediction in this population are limited by low accuracy, a narrow range of considered risk factors, and a lack of interpretability. This study aims to develop an interpretable machine learning model using the CNN-SVM algorithm, integrated with interpretability techniques, for individualized osteoporosis risk assessment in RA patients. The model specifically focuses on the osteopenia stage, which has been overlooked in previous research, to better capture the different risk factors involved in the progression of osteoporosis in RA patients.

**Methods:**

We recruited 314 RA patients from the Department of Rheumatology and Immunology. Participants were categorized into osteoporosis, osteopenia, and normal groups based on lumbar spine or hip bone mineral density (BMD) T-scores. We constructed ML model to assess osteoporosis using a novel classification algorithm, CNN-SVM, and employed SHapley Additive exPlanations (SHAP) and Sankey diagram to investigate significant risk factors, rank risk factor contributions, and provide individualized feature contribution explanations.

**Results:**

A total of 16 candidate variables were included, and three classification models were constructed to predict osteoporosis versus osteopenia, osteoporosis versus normal, and osteopenia versus normal. The AUC values for the models were 0.83, 0.93, and 0.74, respectively. Feature importance analysis using SHAP identified several key predictors. Factors such as Vitamin D supplements, Synovitis in Both Knees, and gender were crucial for distinguishing normal from osteopenia. For differentiating osteoporosis, Alendronate Sodium, weight, and age consistently ranked as highly influential features across different comparisons. Feature importance analysis was performed, ranking risk factors and providing individualized explanations of feature contributions.

**Conclusions:**

The developed interpretable ML model shows promise for screening osteoporosis risk in patients with RA. Its ability to identify individual risk factors highlights its potential to facilitate personalized prevention and management strategies, pending further validation.

**Supplementary Information:**

The online version contains supplementary material available at 10.1186/s12891-026-09526-1.

## Introduction

Rheumatoid arthritis (RA) is a chronic inflammatory joint disease that can lead to cartilage and bone damage, as well as disability [1]. Osteoporosis is a skeletal disorder characterized by reduced bone mass, deterioration of bone tissue microarchitecture, and a decline in bone quality. These changes increase bone fragility and the risk of fractures [2]. Osteoporosis is a major health issue in RA patients, with its prevalence significantly higher compared to the general population [3]. Both localized and systemic bone loss are major extra-articular complications of RA, not only increasing the risk of fractures but also impairing functional capacity, quality of life, and life expectancy [4]. Therefore, identifying high-risk individuals with osteoporosis among RA patients is crucial for alleviating the disease burden.

Clinical assessment tools for osteoporosis, such as the Fracture Risk Assessment Tool (FRAX) [5], Simple Calculated Osteoporosis Risk Estimation (SCORE) [6], Osteoporosis Risk Assessment Instrument (ORAI) [7], Osteoporosis Index of Risk (OSIRIS) [8], and Osteoporosis Self-assessment Tool (OST) [9], have been developed to identify patients at higher risk of osteoporosis [10]. The FRAX tool [5] is applicable to patients aged 40–90 years and incorporates factors such as age, gender, weight, height, and clinical risk factors including previous fractures, parental hip fractures, long-term corticosteroid use, rheumatoid arthritis, smoking, excessive alcohol consumption, secondary osteoporosis (with a yes/no response), and optionally, femoral neck BMD values. SCORE [6] is primarily used for postmenopausal women and screens for osteoporosis risk using simple indicators such as age, weight, and estrogen use. ORAI [7] assesses osteoporosis risk based on age, weight, and estrogen use, suitable for primary screening. OSIRIS [8] is mainly used for screening women with bone density tests and evaluates risk using age, weight, menopausal status, and previous fractures. OST [9] is a rapid screening tool for osteoporosis risk in Asian populations based on age and weight. Among these, only FRAX includes rheumatoid arthritis as a risk factor. However, FRAX does not consider disease duration and disease activity, which are factors that influence fracture risk [11]. It has been shown that FRAX overestimates fracture risk in RA patients, likely due to the increased mortality rate in both RA patients and those using glucocorticoids [12]. For instance, a meta-analysis highlights that RA patients are influenced not only by traditional osteoporosis risk factors but also by RA-specific factors, such as disease duration, disease activity, and Health Assessment Questionnaire (HAQ) score, which significantly increase the risk of fractures [13]. In light of this, it is crucial to enhance prediction models by incorporating additional predictive indices based on existing frameworks, thereby improving both prediction accuracy and discriminatory power.

Artificial intelligence and machine learning techniques have garnered continued attention for medical risk prediction [14]. Recent studies have demonstrated the robust applicability of algorithms such as Random Forest, Support Vector Machines (SVM), and discriminant analysis in predicting diverse conditions, ranging from hepatic encephalopathy complications and breast cancer to coronary heart disease [15–17]. In the specific domain of osteoporosis, algorithms like K-NN have shown promise in risk prediction [18], while others have utilized ML outputs to propose personalized sports protocols [19].

However, applying these techniques to RA-associated osteoporosis presents unique challenges. While recent studies have identified important predictors, they retain critical methodological shortcomings. For instance, Yan et al. [20] developed a nomogram achieving good discrimination; however, their model relied on a binary classification (Osteoporosis vs. Non-Osteoporosis), which oversimplifies the disease spectrum by failing to distinguish the clinically critical pre-osteoporotic stage of osteopenia. Similarly, while Chen et al. [21] and Lee et al. [22] employed advanced ensemble algorithms like XGBoost and LightGBM to handle high-dimensional data, their approaches effectively function as 'black boxes.' These models provide global feature importance but lack granular interpretability, failing to elucidate how specific risk factors drive predictions for individual patients—a crucial requirement for building clinical trust and facilitating personalized intervention [10, 23].

To bridge these gaps, this study was designed to achieve two primary objectives directly addressing the limitations of prior research. First, to overcome the oversimplification of binary models, we developed a hybrid Convolutional Neural Network-Support Vector Machine (CNN-SVM) architecture capable of accurately distinguishing between three bone density states, with a specific focus on the challenging separation of adjacent states (e.g., Normal vs. Osteopenia). Second, to address the 'black box' issue, we integrated an Explainable AI (XAI) framework using SHAP. This approach aims to move beyond static risk scores by visualizing dynamic, stage-dependent risk factor hierarchies, thereby providing the transparent, individualized insights necessary for personalized preventative strategies in RA patients.

## Materials and methods

### Data collection and preprocessing

This study included 314 RA patients, all of whom were recruited from the inpatient department of the Second Xiangya Hospital of Central South University and diagnosed with RA according to the 2009 ACR/EULAR classification criteria. The sample consisted of both male and female patients, with females comprising 69.75%. Participants' ages ranged from 22 to 86 years, with a mean age of approximately 61 years. The use of dual-energy X-ray absorptiometry (DXA) to measure BMD is the standard diagnostic tool for osteoporosis. Lumbar spine and hip DXA scans were performed using a DXA Hologic scanner (ASY-00409; Hologic, Inc., Bedford, MA), and the equipment was calibrated automatically. The T-value was calculated according to the BMD reference value of healthy adults of the same sex and nationality [24]. Based on World Health Organization criteria [25] (T-score ≥ − 1.0 for normal, − 2.5 < T-value < −1.0 for osteopenia, and T-value ≤ − 2.5 for osteoporosis), we classified patients based on their most severe reading from either the lumbar spine or hip. This resulted in three final groups: osteoporosis (103, 32.8%), osteopenia (130, 41.4%), and normal (81, 25.8%).

While this cohort of 314 patients represents a modest sample size, it constitutes a strictly curated clinical dataset ensuring high data completeness and reliability. We acknowledge that this sample size, drawn from a single inpatient center, may limit the immediate generalizability of the model to broader, heterogeneous populations or outpatient settings where disease severity profiles may differ. To address this challenge and mitigate the risk of overfitting to this specific cohort, our modeling approach incorporated rigorous regularization strategies, including architectural constraints (Dropout, Batch Normalization) and Early Stopping (detailed in Section Model training and evaluation). Consequently, this study is framed as an exploratory analysis identifying high-priority response signatures, paving the way for future validation in larger, multi-center cohorts.

From an initial pool of 81 potential variables, a final set of 16 predictors was derived through a two-stage data-driven feature selection process. First, a filtering step was conducted to remove features with low statistical association to the outcome. We calculated the Pearson's correlation coefficient between each predictor and the ordinal outcome variable (coded as 0 = Normal, 1 = Osteopenia, 2 = Osteoporosis), excluding all features with an absolute correlation coefficient |r|< 0.1.

Subsequently, the remaining subset of variables was evaluated using a wrapper-based selection method. We employed recursive feature elimination with cross-validation (RFECV) with a Random Forest classifier as the estimator. This process iteratively trained the model, pruned the least important feature(s) based on model-derived importance, and selected the feature subset that maximized the average cross-validated AUC score across our three classification tasks. This approach resulted in the final 16-predictor set used for all subsequent model development.

**Fig. 1:**
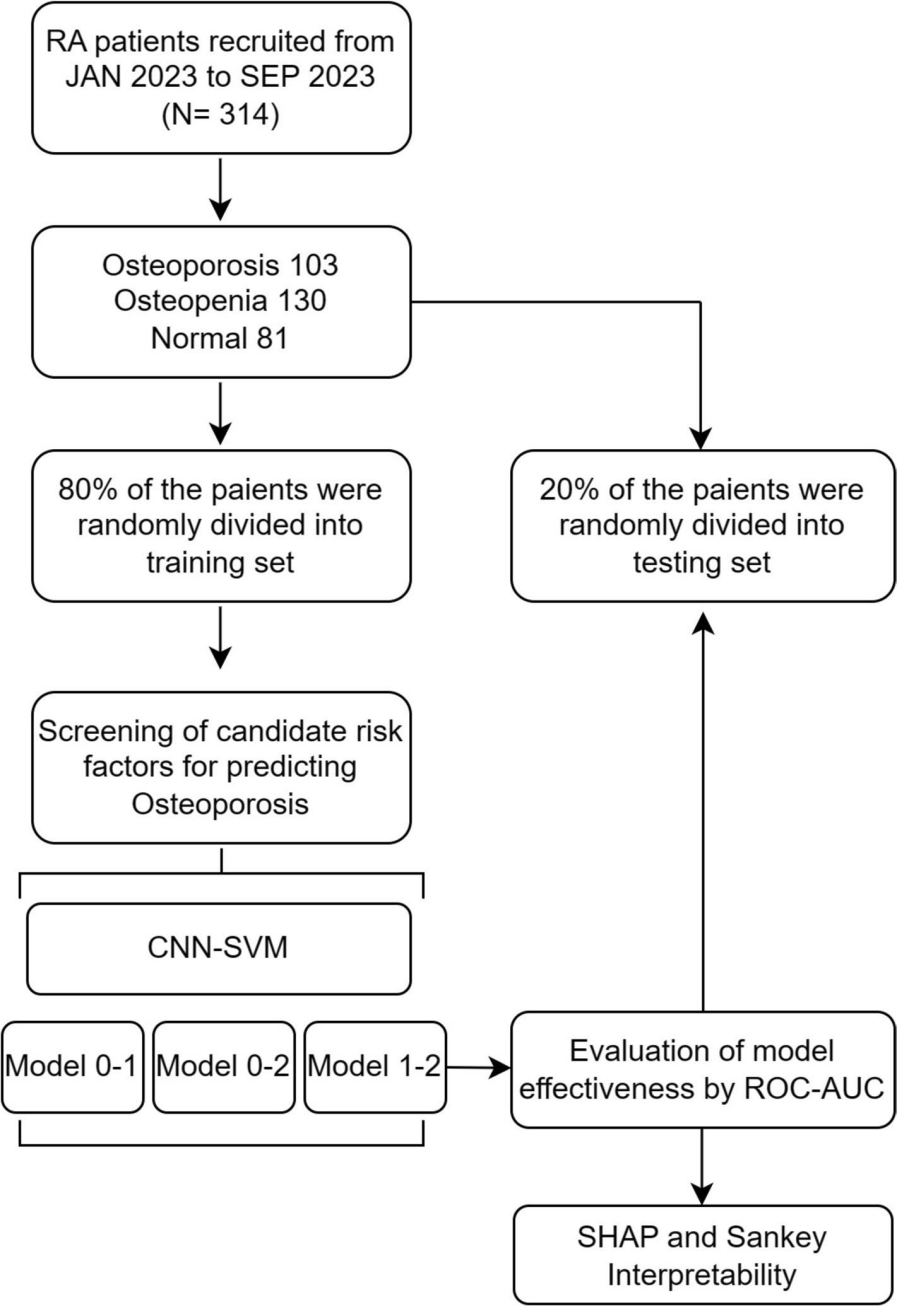
Flow diagram of study population and data process

A key criterion during this selection process was to identify a subset of features that had complete data (no missing values) across the entire final cohort (N = 314), thus obviating the need for data imputation in the final modeling stage. For the categorical variables, we employed label encoding. It is important to clarify that this method does not introduce arbitrary bias in our specific feature set. The majority of our categorical predictors are binary (e.g., Gender, Fracture History, Medication Use), where label encoding (0/1) is mathematically equivalent to binary encoding and introduces no artificial ordinality. The remaining categorical variable, 'Daily Physical Activity Level,' is inherently ordinal (Light, Medium, Heavy). For this variable, label encoding is methodologically appropriate as it preserves the rank-order relationship essential for the SVM classifier to interpret increasing levels of activity. Thus, this encoding strategy aligns with the data structure without compromising the model's validity. All 16 features were then standardized using a StandardScaler prior to being input into the CNN-SVM model. We randomly selected 80% of the patients for the training set to develop the models, while the remaining 20% were allocated to the test set for validation, as shown in Fig. 1.

### Model selection and rationale

The selection of the hybrid CNN-SVM was informed by a preliminary benchmarking analysis against ten conventional machine learning algorithms (Supplementary Table S1). This comparison revealed a critical performance gap: while many algorithms performed well on the well-differentiated Normal vs. Osteoporosis task (AUCs up to 0.935), their efficacy was substantially limited on the more challenging task of separating adjacent states, particularly Normal vs. Osteopenia (Task 0–1), where the CNN-SVM achieved a superior AUC of 0.833.

Theoretically, this empirical advantage stems from the architecture's synergistic design. The one-dimensional CNN component functions as an automated deep feature extractor, adept at learning hierarchical representations and capturing complex non-linear patterns and feature interactions from the clinical data vector. The resulting 64-dimensional abstract feature set is then classified by a linear Support Vector Classifier (SVC), which provides a robust maximal-margin decision boundary. This hybrid approach, combining deep feature engineering with a powerful classifier, is thus particularly well-suited for this nuanced clinical problem, justifying its selection as our primary model for in-depth analysis and interpretation.

From a clinical perspective, the rationale for this hybrid architecture can be understood as mirroring a two-stage diagnostic process. Functionally, the CNN component serves as a deep feature extractor, capable of identifying complex, non-linear interactions among risk factors—much like a clinician synthesizing subtle combinations of symptoms and history that standard scoring systems might overlook. Subsequently, the SVM component acts as a robust classifier, utilizing these synthesized features to determine the optimal decision boundary for patient stratification. This synergy is particularly advantageous for this study, as it combines the pattern-recognition depth of deep learning with the classification stability of SVMs in moderate-sized clinical datasets.

### Model training and evaluation

Based on the model selection process, we proceeded with the hybrid CNN-SVM model (Supplementary Table S1). Three separate models were trained for the binary classification tasks: normal vs. osteopenia, normal vs. osteoporosis, and osteopenia vs. osteoporosis. The model architecture consists of two stages: a deep feature extractor followed by a linear classifier.

The feature extractor is a one-dimensional Convolutional Neural Network (CNN). Its architecture comprises two sequential blocks. The first block contains two Conv1D layers with 32 filters each, a kernel size of 3, and ReLU activation, followed by MaxPooling1D. The second block consists of two Conv1D layers with 64 filters each, also with a kernel size of 3 and ReLU activation, followed by AveragePooling1D. To prevent overfitting, BatchNormalization and Dropout (rate = 0.4) were applied after each block. The output of the convolutional blocks is flattened and passed through two Dense layers (128 and 64 units, respectively) with ReLU activation and Dropout (rate = 0.5).

The 64-dimensional output vector from the final dense layer of the CNN serves as the extracted features. These features are then used to train a SVC with a linear kernel. This hybrid approach, where the CNN acts as a sophisticated feature extractor for the SVM, is designed to capture complex non-linear patterns while maintaining a robust classification boundary.

The CNN component was trained for up to 200 epochs with a batch size of 32, using the Adam optimizer (learning rate = 0.001) and categorical cross-entropy loss. We employed two key strategies for robust training: class_weight = 'balanced' was used to address the class imbalance in the training data, and an EarlyStopping callback (monitoring val_loss with a patience of 30) was used to select the best model epoch and prevent overfitting.

We chose AUC as the primary metric because it is threshold-independent and robust to class imbalance, providing a comprehensive assessment of the model's overall discriminative power, which is particularly valuable for clinical screening applications. The calculation method is shown in Eq. (1) and (2). Confusion matrices provide detailed insights into model predictions by displaying true positives, true negatives, false positives, and false negatives, allowing the calculation of accuracy, precision, recall, and F1 score. Together, these metrics offer a comprehensive evaluation of a model's performance, highlighting both overall effectiveness and specific strengths and weaknesses. The ROC curves and corresponding AUC values of the optimized ML models highlight the models' predictive capabilities. The confusion matrix further underscores the predictive prowess of these models.

1 $${\mathrm{AUC}}={\sum }_{i=1}^{n-1}\left(\frac{{\mathrm{TPR}}_{i+1}+{\mathrm{TPR}}_{i}}{2}\right)\left({\mathrm{FPR}}_{i+1}-{\mathrm{FPR}}_{i}\right)$$

2 $${\mathrm{TPR}}=\frac{\mathrm{TP}}{{\mathrm{TP}}+{\mathrm{FN}}}$$

3 $${\mathrm{FPR}}=\frac{\mathrm{FP}}{{\mathrm{FP}}+{\mathrm{TN}}}$$

### Explainable machine learning ensembles

SHAP values were utilized to evaluate the influence and significance of each input variable on the model’s predictions. SHAP, a game-theoretic approach, interprets the outputs of any machine learning model by applying classical shapley values from cooperative game theory [21], thereby optimally attributing credit to individual feature contributions. This method offered valuable insights into which factors among RA patients played the most crucial roles in predicting osteoporosis risk. The SHAP calculation method is shown in Eq. (4), where: ${\upphi }_{i}$ represents the marginal contribution of feature i to the model prediction value after adding it to subset S, $N$ represents the set of all features, S represents a subset of features, excluding feature i, $\left|S\right|$ and $\left|N\right|$ represent the the size of subset S and the total number of features respectively, v(S) represents the model’s prediction value on subset S, $\mathrm{v}\left(\mathrm{S}\cup \{\mathrm{i}\}\right)-\mathrm{v}\left(\mathrm{S}\right)$ represents the marginal contribution of adding feature i to subset S on the model’s prediction value.

4 $${\phi }_{i}={\sum }_{S\subseteq N\setminus \{i\}}\frac{\left|S\right|!\left(\left|N\right|-\left|S\right|-1\right)!}{\left|N\right|!}\left[v\left(S\cup \{i\}\right)-v\left(S\right)\right]$$

There exists a correlation between different feature columns and the sample label column. If the correlation between feature columns and the sample label column is not significant, it will affect the model's discriminative ability. Therefore, dimensionality reduction is necessary to extract highly correlated features for model training.

We classified the respondents into normal, osteopenia, or osteoporosis groups based on their lumbar spine or hip BMD results, and used these classifications as sample labels. We determined the predictors for osteoporosis by calculating the correlation between features and class labels, combined with recursive feature elimination. This process allowed us to remove features with low correlation(correlation < 0.1) and non-linear characteristics, ultimately identifying the most relevant predictors based on the model's requirements. The final features used for model training included 6 numerical features and 10 categorical features.

### Statistic analysis

Statistical analyses were performed using Python (version 3.9, Python Software Foundation). Categorical variables were presented as frequencies (or percentages) and compared using the chi-square test or Fisher’s exact test. To check the normality of continuous data, the Kolmogorov–Smirnov-Lilliefors (K-S-L) test was used. For variables that did not conform to normal distribution, the Wilcoxon rank-sum test was employed. Statistical significance was defined as a p-value < 0.05.

## Results

### Baseline characteristics

The baseline characteristics of the respondents from the preprocessed data sets are presented in Table 1. Among the 314 respondents, the average age was approximately 61 years, with 69.75% being Female. From the last two rows of Table 1, it can be seen that, for both Lumbar_Spine_Osteoporosis and Hip_Osteoporosis, osteopenia accounted for the highest proportion among the three categories.

**Table 1:** General characteristics of respondents

Variable	Normal	Osteopenia	Osteoporosis	*P* value
Gender, n(%)				< *0.05*
Female	*49 (60.49)*	*90 (69.23)*	*80 (77.67)*	
Male	*32 (39.51)*	*40 (30.77)*	*23 (22.33)*	
Age (years), mean(SD)	*58.74 (10.71)*	*60.31 (11.59)*	*64.94 (8.11)*	< *0.001*
BMI	*22.81 (3.39)*	*22.39 (3.39)*	*20.85 (3.57)*	< *0.001*
Fracture_History, n(%)				*0.2845*
Yes	*10 (12.35)*	*15 (11.54)*	*19 (18.45)*	
No	*71 (87.65)*	*115 (88.46)*	*84 (81.55)*	
Tender_Joint_Count, mean(SD)	*12.72 (10.06)*	*10.44 (9.22)*	*10.46 (8.33)*	*0.1603*
Swollen_Joint_Count, mean(SD)	*10.01 (8.60)*	*7.57 (8.06)*	*9.16 (7.76)*	*0.0849*
DAS28_Score, mean(SD)	*5.95 (1.63)*	*5.54 (1.59)*	*5.85 (1.40)*	*0.1249*
Vitamin D Level, mean(SD)	*65.16 (16.81)*	*62.63 (17.66)*	*57.50 (17.67)*	< *0.05*
Vitamin D Supplements, n(%)				< *0.001*
Yes	*49 (60.49)*	*103 (79.23)*	*90 (87.38)*	
No	*32 (39.51)*	*27 (20.77)*	*13 (12.62)*	
Alcohol_Consumption_History				*0.0613*
Yes	*11(13.58)*	*8(6.15)*	*5(4.85)*	
No	*70(86.42)*	*122(93.85)*	*98(95.15)*	
Alendronate_Sodium				< *0.001*
Yes	*3(3.7)*	*19(14.62)*	*41(39.81)*	
No	*78(96.3)*	*111(85.38)*	*62(60.19)*	
Golimumab				< *0.05*
Yes	*0(0)*	*9(6.92)*	*14(13.59)*	
No	*81(100.00)*	*121(93.08)*	*89(86.41)*	
Tofacitinib				*0.1601*
Yes	*28(34.57)*	*33(25.38)*	*23(22.33)*	
No	*53(65.43)*	*97(74.62)*	*80(77.67)*	
Daily Physical Activity Level				< *0.001*
Light	*46(56.79)*	*47(36.15)*	*27(26.21)*	
Medium	*13(16.05)*	*25(19.23)*	*18(17.48)*	
Heavy	*22(27.16)*	*58(44.62)*	*58(56.31)*	
Synovitis in Both Knees				*0.1600*
Yes	*61(75.31)*	*86(66.15)*	*64(62.14)*	
No	*20(24.69)*	*44(33.85)*	*39(37.86)*	
Synovitis in Both Elbows				*0.0629*
Yes	*11(13.58)*	*12(9.23)*	*4(3.88)*	
No	*70(86.42)*	*118(90.77)*	*99(96.12)*	
Lumbar_Spine_Osteoporosis, n(%)	*81(25.80)*	*130(41.40)*	*103(32.80)*	
Hip_Osteoporosis, n(%)	*76(24.20)*	*178(56.69)*	*60(19.11)*	

*SD* Standard Deviation

### Model performances

We comprehensively evaluated the performance of the final CNN-SVM model on the held-out test set across the three distinct classification tasks. The model demonstrated strong and statistically significant discriminative ability for all tasks (all *p* < 0.002 compared to random chance).

**Fig. 2:**
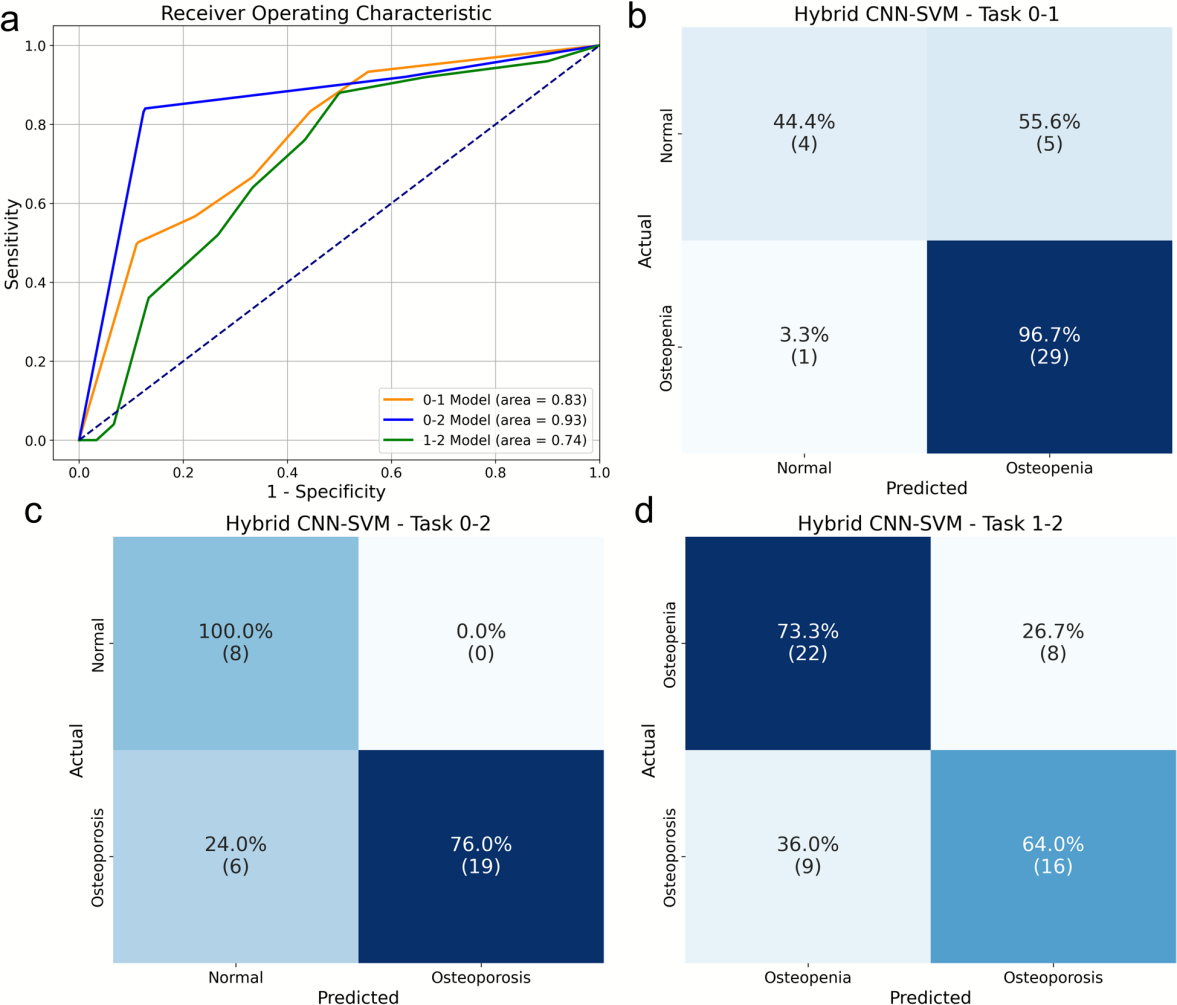
Results for osteoporosis prediction models in RA patients. **a** ROC curves of the DL models. **b** The confusion matrix of the normal versus osteopenia. **c** The confusion matrix of the normal versus osteoporosis. **d** The confusion matrix of the osteopenia versus osteoporosis

As detailed in Table 2 and illustrated by the ROC curves in Fig. 2a, the model achieved an AUC of 0.93 (95% CI, 0.81–1.00) for the well-separated Normal vs. Osteoporosis task. For the more challenging adjacent-state tasks, it yielded an AUC of 0.83 (95% CI, 0.67–0.97) for Normal vs. Osteopenia and an AUC of 0.74 (95% CI, 0.59–0.87) for Osteopenia vs. Osteoporosis. The performance on the Normal vs. Osteopenia task was notably superior to that of the ten conventional machine learning algorithms benchmarked in our preliminary analysis (see Supplementary Table S1), which justified the selection of the CNN-SVM architecture.

**Table 2:** Detailed performance metrics of the CNN-SVM model on the test

Task	Class	Preision	Recall	F1-Score	Accuracy	AUC(95% CI)	*p*-value
0–1 (Normal vs Osteopenia)	Normal	0.80	0.44	0.57	0.85	0.83 (0.67–0.97)	0.001
	Osteopenia	0.85	0.97	0.91			
0–2 (Normal vs. Osteoporosis)	Normal	0.57	1.00	0.73	0.82	0.93 (0.81–1.00)	< 0.001
	Osteoporosis	1.00	0.76	0.86			
1–2 (Osteopenia vs. Osteoporosis)	Osteopenia	0.71	0.73	0.72	0.69	0.743 (0.59–0.87)	0.002
	Osteoporosis	0.67	0.64	0.65

The confusion matrices (Fig. 2b-d) and class-specific metrics in Table 2 offer further insights into the model's classification behavior. For the Normal vs. Osteoporosis task (Fig. 2c), the model achieved a perfect recall of 1.00 for the Normal class, indicating that no healthy individuals were misclassified as having osteoporosis, a critical feature for avoiding unnecessary alarm.

More revealing are the results for the most challenging task, differentiating Normal from Osteopenia (Fig. 2b). Here, the model exhibited a pronounced asymmetric error profile: its primary misclassification was labeling Normal patients as having Osteopenia (a 55.6% error rate for the Normal class), while very rarely misclassifying at-risk Osteopenia patients as Normal (a low 3.3% error rate). From a clinical screening perspective, this "fail-safe" tendency can be interpreted as a desirable feature. It prioritizes high sensitivity for the 'at-risk' osteopenia group at the expense of specificity for the healthy group, a common and often prudent trade-off where the clinical cost of a false negative (missing a patient in the early stages of bone loss) is significantly higher than that of a false positive (recommending lifestyle monitoring for a healthy individual). A more detailed discussion of this error profile is presented in the Discussion section.

### Response signatures

To elucidate the factors driving the model's predictions, we employed two primary explainability techniques, SHAP and Sankey diagrams, on the best-performing CNN-SVM model.

**Fig. 3:**
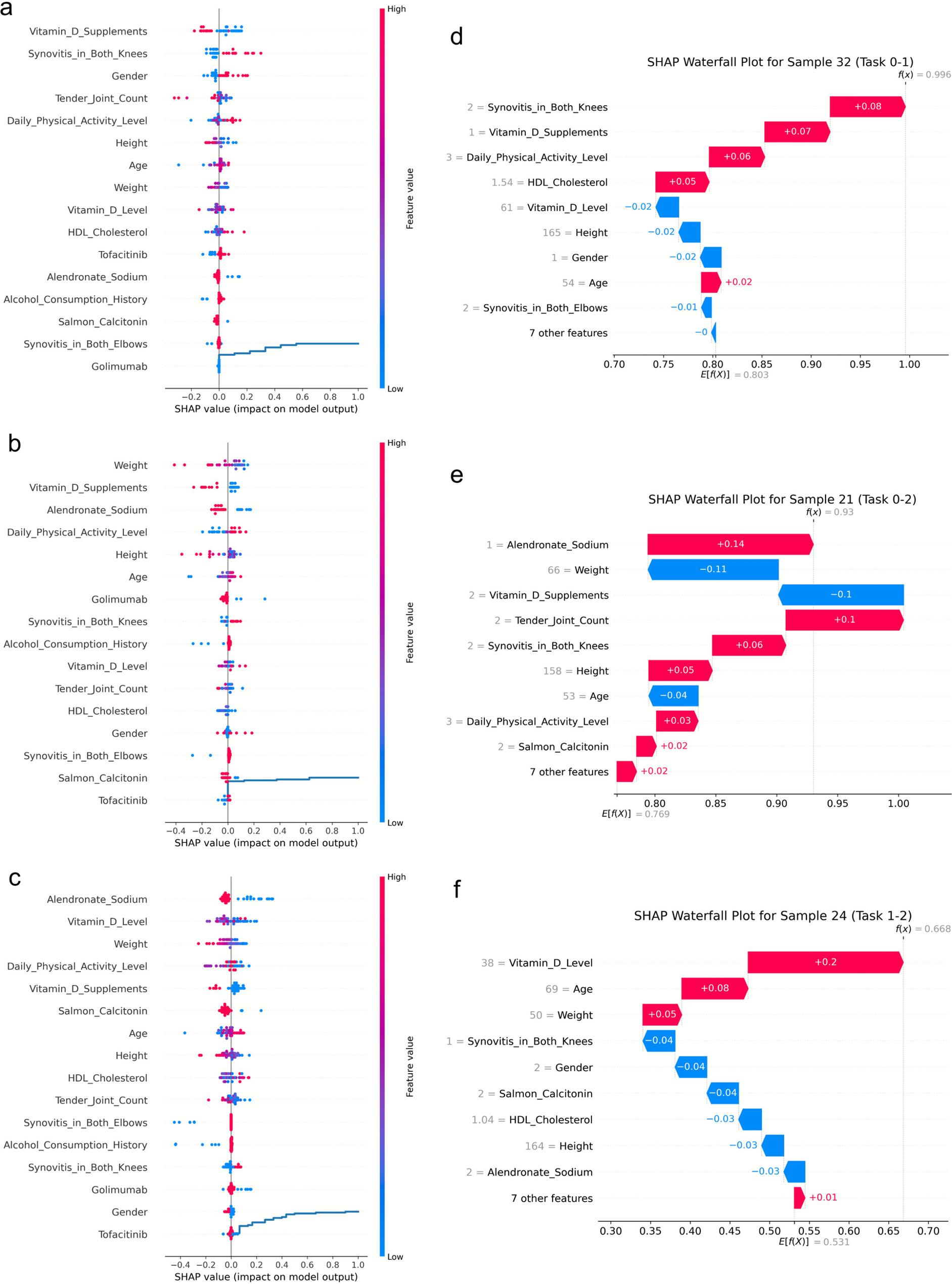
Interpretable illustration for osteoporosis prediction models. **a** SHAP beeswarm plot for normal versus osteopenia. **b** SHAP beeswarm plot for normal versus osteoporosis. **c** SHAP beeswarm plot for osteopenia versus osteoporosis. **d** SHAP waterfall plot for normal versus osteopenia. **e** SHAP waterfall plot for normal versus osteoporosis. **f** SHAP waterfall plot for osteopenia versus osteoporosis

First, SHAP analysis was used to quantify feature importance for each of the three classification tasks (Fig. 3a-c). The SHAP summary plots rank features based on their mean absolute impact on the model's output. A key finding is that the hierarchy of risk factors is dynamic, shifting depending on the classification task. For the Normal vs. Osteopenia task (Fig. 3a), the top-ranked predictors were Vitamin D Supplements, Synovitis in Both Knees, and Gender. In contrast, for the Normal vs. Osteoporosis task (Fig. 3b), factors like Weight, Vitamin D Supplements, and Alendronate Sodium were most important. For the Osteopenia vs. Osteoporosis comparison (Fig. 3c), Alendronate Sodium, Vitamin D Level, and Weight were the most significant predictors.

**Fig. 4:**
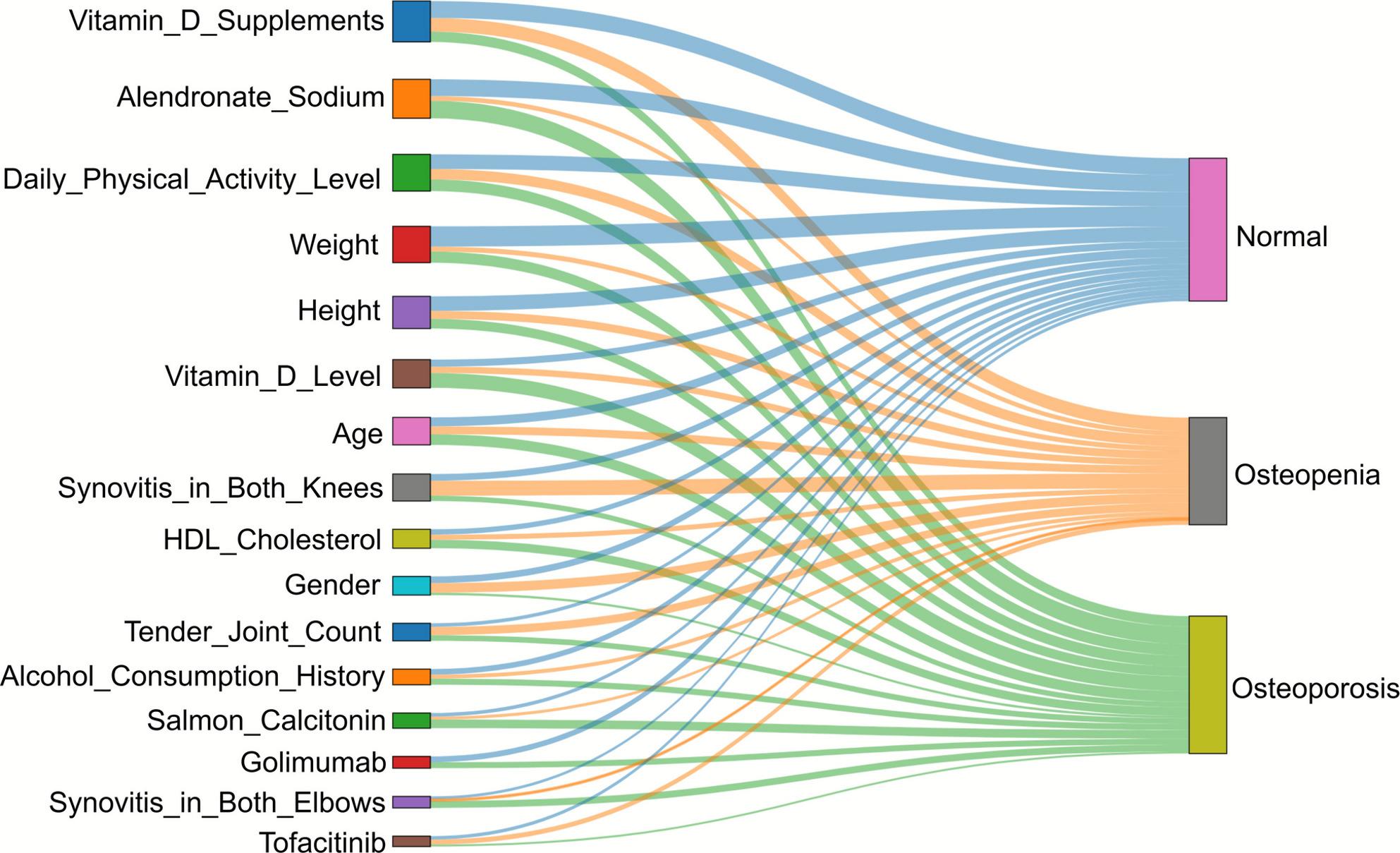
Sankey diagram of individualized model evaluation

Second, to provide a holistic visualization of how these features collectively contribute to patient stratification, we generated a Sankey diagram (Figure 4). This diagram is a conceptual tool built upon the aggregated SHAP values, illustrating the overall flow of feature contributions from the 16 predictors (left) towards the three predicted states (right). The width of each flow is proportional to the feature's aggregate importance (mean absolute SHAP value) in contributing to the model's overall predictions across the tasks. By observing the flows, the diagram intuitively confirms the dynamic, stage-dependent nature of the risk factors. For example, prominent flows from Age, Weight, and Alendronate Sodium can be seen directed towards the Osteoporosis node, visually reinforcing their dominant role in identifying established disease. Conversely, flows from Vitamin D Supplements and Synovitis in Both Knees show strong contributions to the stratification between the Normal and Osteopenia nodes, aligning with their high importance in the SHAP analysis for that specific task (Task 0-1). This visualization thus provides a powerful, conceptual map of the stage-dependent interplay of risk factors learned by our model.

Finally, to demonstrate the model's utility for personalized risk assessment, we generated SHAP waterfall plots for representative individual samples from the test set (Figure 3d-f). These plots decompose a single prediction, showing precisely how each feature value for a given patient, such as the presence of synovitis or the use of specific medications—contributes to pushing the risk score higher (in red) or lower (in blue) from a baseline, leading to the final classification.

Table 3 illustrates the differences in osteoporosis prediction factors across different studies, highlighting the importance of using risk factors specific to certain patient populations (e.g., RA patients) for prediction. In Non-RA studies, key osteoporosis risk factors identified align with traditional clinical factors. For instance, age, weight, and BMI rank highly across most studies, including those by Erjiang et al. [26], Park et al. [27], and Bui et al. [28]. Additionally, Bui et al. [28] highlighted bisphosphonate use in the top three, while Erjiang et al. [26] ranked sodium fluoride use in the top five, emphasizing the role of medication in osteoporosis risk.

**Table 3:** Comparative analysis of top 10 features in osteoporosis prediction studies

	**Non-RA**			**RA**					
Ranking	**Erjiang et al.** [26]	**Park et al. ** [27]	**Bui et al. ** [28]	**Shodikulova et al. ** [29]	**Hu et al. ** [30]	**Yan et al. ** [20]	**Chen et al. ** [21]	**Lee et al. ** [22]	**Our study**
1	*Age*	*Age*	*Age*	*Joint Activity*	*Age*	*Age*	*Serum Selenium*	*Body mass index*	*Weight*
2	*Weight*	*Menopause*	*Weight*	*Immunologic Variants*	*Body Mass Index*	*Disease Course*	*Bone Mineral Density*	*Age*	*Vitamin D Supplements*
3	*Bisphosphonate use*	*Alkaline phosphatase*	*Height*	*Age*	*Serum 25-hydroxyvitamin D3 Level*	*DAS28*	*Rheumatoid Factor*	*Menopause*	*Alendronate Sodium*
4	*Body Mass Index*	*Weight*	*Uric acid*	*Glucocorticoids*	*Tumor Necrosis Factor Inhibitor Usage*	*Anti-Cyclic Citrullinated Peptide Antibody*	*High-sensitivity C-Reactive Protein*	*Waist circumference*	*Daily physical activity level*
5	*Denosumab use*	*Forced vital capacity*	*Calcium*	*Hypocalcemia*	*Serum Uric Acid Level*	*7-Joint Ultrasonic Bone Erosion*	*Erythrocyte Sedimentation Rate*	*Hip circumference*	*Height*
6	*Estrogen use*	*Sobriety*	*Cholesterol*	*Family History of Bone Fractures*	*Disease Duration*	*/*	*History of Long-term Glucocorticoid Use*	*Surgery for RA*	*Age*
7	*Chronic Respiratory* *Disease*	*Aspartate* *aminotransferase*	*Creatinine*	*Menopause*	*/*	*/*	*RA Duration*	*Monthly income*	*Golimumab*
8	*Osteopenia*	*Diastolic blood pressure*	*Free thyroxine level*	*Back Pain and Joint Deformation*	*/*	*/*	*Family History of Osteoporosis*	*Age of diagnosis*	*Synovitis in Both Knees*
9	*Height loss*	*Riboflavin intake*	*Glucose*	*Physical Activity and Nutrition*	*/*	*/*	*Smoking History*	*Health assessment questionnaire*	*Alcohol Consumption History*
10	*Hormonal Therapy*	*Weight control by exercise*	*HbA1c*	*/*	*/*	*/*	*Body Mass Index*	*Number of swollen joints*	*Vitamin D level*

In contrast, in RA studies, key features identified differ significantly from those in Non-RA populations, reflecting the unique impact of rheumatoid arthritis, a chronic autoimmune disease, on bone metabolism. RA studies typically include disease-specific factors such as disease duration [21], DAS28 [20], 25-hydroxyvitamin D3 levels [29], and medication use such as corticosteroid use [21] and tumor necrosis factor inhibitors [30], all of which consistently rank highly in RA cohorts. Lee et al. [22] also included unique factors like RA surgery history and monthly income. Our study confirms the importance of traditional factors such as age and weight, as well as lifestyle-related factors like alcohol consumption history and Vitamin D Supplementation. Additionally, we introduced RA-related features, such as synovitis in both knees, Alendronate Sodium, and Golimumab, as significant risk factors. Our findings, alongside comparisons with previous studies, provide a comprehensive framework for assessing osteoporosis in RA patients.

## Discussions

Osteoporosis reduces bone density and increases susceptibility to fractures, posing a significant health issue for patients with rheumatoid arthritis (RA). Notably, osteoporosis often presents no obvious symptoms until fractures occur, highlighting the urgency of early detection and preventive strategies [31]. Early interventions, including lifestyle modifications and pharmacological treatments, have proven to be much more effective when osteoporosis is identified at an early stage [32].

Our study leveraged an explainable AI framework to move beyond simple risk prediction and elucidate the specific response signatures of osteoporosis in RA. A key finding from our SHAP analysis (Fig. 3) is that the hierarchy of risk factors is dynamic, shifting in importance depending on the stage of bone loss. This provides a more nuanced understanding compared to traditional models. For the crucial early transition from normal to osteopenia (Task 0–1), lifestyle and inflammatory factors such as Vitamin D Supplements and Synovitis in Both Knees were paramount. This emphasis on synovitis, a direct marker of joint inflammation, supports the hypothesis that RA-specific inflammatory processes are key triggers in the initiation of bone loss, a factor not as prominently featured in other RA studies [21].

In contrast, when predicting established osteoporosis from a normal state (Task 0–2) or its progression from osteopenia (Task 1–2), the feature hierarchy shifted. Here, factors related to long-term bone health, established risk, and treatment history, such as Weight, Age, and the use of Alendronate Sodium, became more dominant. This aligns with findings from both Non-RA [26–28] and RA-specific [20–22, 29, 30] studies, which consistently identify age and weight as major predictors. Our model further highlighted the importance of specific medications like Alendronate Sodium and Golimumab, and lifestyle factors like alcohol consumption history, confirming their roles in the broader context of bone metabolism in RA, consistent with observations by Hu et al. [30] and Chen et al. [21]. This stage-dependent importance of risk factors underscores the value of our three-model approach, offering a more dynamic view of osteoporosis pathogenesis than a single, static model.

Prior to analyzing specific misclassifications, it is crucial to interpret the model's overall discriminative performance across these stages. Our analysis reveals a clear performance gradient consistent with the biological reality of bone disease. The model achieved excellent discrimination (AUC 0.93) when distinguishing clinically distinct states (Normal vs. Osteoporosis), but showed moderate performance (AUC 0.83 and 0.74) for adjacent states. This discrepancy reflects the continuous physiological spectrum of bone density loss, where the boundary between 'high-normal' and 'early-osteopenia' is often defined by marginal differences in T-scores rather than distinct pathophysiological leaps. Clinically, this suggests that while the model is highly effective at flagging established disease, its application in borderline cases (adjacent states) should be interpreted as a probabilistic risk assessment rather than a definitive diagnostic verdict, serving to highlight patients who warrant closer monitoring.

Furthermore, the confusion matrices (Fig. 2b-d) reveal a clinically pragmatic 'fail-safe' classification pattern, particularly in the challenging Normal vs. Osteopenia task. The model exhibited a 55.6% misclassification rate for Normal patients (labeling them as Osteopenia), while maintaining a minimal 3.3% error rate for missing actual Osteopenia cases. To quantify the clinical impact of this trade-off, we assessed the specific interventions triggered by these errors. A false positive (labeling a healthy patient as 'at-risk') results in low-burden, low-risk interventions: specifically, increased clinical vigilance, optimization of dietary calcium and Vitamin D, and lifestyle modifications for fall prevention. These measures typically carry minimal adverse effects. In stark contrast, a false negative (missing a patient with early bone loss) carries a high clinical cost: the loss of a critical therapeutic window to prevent irreversible microarchitectural deterioration, significantly increasing the risk of fragility fractures and subsequent disability. Therefore, the model's bias towards over-estimating risk essentially prioritizes sensitivity, aligning with the preventative rheumatology imperative to minimize missed diagnoses in at-risk populations.

Beyond identifying these risk signatures, the practical implementation of this explainable model in clinical practice warrants consideration. The final 16-predictor model relies on variables (e.g., age, weight, medication history, synovitis assessment) that are largely part of a standard rheumatology consultation, suggesting a low barrier to data acquisition. A potential integration pathway involves embedding the model within an Electronic Health Record (EHR) system as a Clinical Decision Support (CDS) tool. Such a tool could automatically calculate and display risk probabilities for the three bone density states at the point of care. Crucially, its explainability could empower clinicians by providing the "why" behind a risk score, facilitating more informed and personalized conversations with patients about preventive strategies—for instance, by visually emphasizing why synovitis control or Vitamin D supplementation is particularly critical for their individual profile. This transparency is essential for building trust and bridging the gap between prediction and practical intervention.

This study has several limitations that should be addressed in future research.

First, our study is constrained by its single-center, retrospective design and a modest sample size (*N* = 314). Recruiting exclusively from the inpatient department introduced a specific selection bias towards patients with higher disease activity or comorbidities. In terms of real-world applicability, this implies that the model has 'learned' a risk profile characteristic of a moderate-to-severe RA phenotype. Consequently, strong predictors identified here, such as 'Synovitis in Both Knees,' might be less prominent in a general ambulatory (outpatient) population where disease activity is often milder or well-controlled. Therefore, while the current model is well-positioned for risk stratification in hospitalized or high-acuity cohorts, its direct deployment in primary care or general outpatient settings may require recalibration to ensure it remains sensitive to the subtler risk signatures associated with mild RA.

Second, we did not perform subgroup or sensitivity analyses. While our model demonstrates good overall performance, its fairness and robustness across different demographic strata (e.g., by gender, age groups, or disease duration) have not been formally assessed. Such analyses were precluded by the current sample size.

Third, modeling the specific impact of pharmacological treatments remains a complex challenge. While our model retained medications like Golimumab and Tofacitinib, their contribution to the final decision (SHAP values) appeared relatively small. This likely reflects statistical constraints and feature resolution rather than a lack of biological effect. Specifically, the low prevalence of usage for these targeted therapies within our cohort limited the model's ability to learn robust associations compared to highly prevalent factors like age or weight. Furthermore, our current binary encoding (presence/absence) fails to capture cumulative exposure (dosage × duration), which is a critical determinant of drug-induced bone modulation. Future studies should aim to incorporate longitudinal cumulative dosage data and recruit larger cohorts. This would allow for a more granular analysis capable of statistically disentangling the direct bone-modifying effects of these medications from the underlying high disease severity for which they are prescribed.

Fourth, our data preprocessing included the use of label encoding for categorical variables. This method introduces an arbitrary ordinal relationship between categories, which may not be optimal for the SVM component of our architecture and could potentially influence the CNN's feature extraction process. Future work should employ one-hot encoding to eliminate this potential source of bias.

To mitigate these limitations, future research should prioritize a multicenter, prospective cohort design that enrolls both inpatient and outpatient participants. This approach would not only enhance sample size and representativeness but would also allow for a deeper understanding of causal relationships. Such larger datasets would also enable the crucial subgroup analyses needed to ensure model equity and robustness.

## Conclusion

This study successfully developed and validated an interpretable, hybrid CNN-SVM framework for personalized osteoporosis risk assessment in RA patients, uniquely addressing the often-overlooked osteopenia stage through a multi-class classification approach. By leveraging explainable AI, we moved beyond simple risk prediction to elucidate the dynamic, stage-dependent hierarchy of risk factors, demonstrating that predictors for the initial transition to osteopenia (e.g., synovitis, Vitamin D supplements) differ from those for established osteoporosis (e.g., age, weight, Alendronate Sodium).

The clinical implications of these findings are significant. The model's ability to identify stage-specific drivers can guide more personalized and timely interventions; for instance, prioritizing aggressive inflammation control with synovitis to prevent early bone loss, versus focusing on fall prevention and bone-resorptive therapies in patients progressing towards osteoporosis. The transparency afforded by SHAP analysis provides a foundation for creating clinical decision support tools that not only flag high-risk individuals but also explain why a patient is at risk, facilitating shared decision-making and patient education.

Moving forward, the refinement of this model requires specific advancements in data collection and feature integration. Future research should prioritize a multi-center, prospective design that encompasses diverse RA populations, including ambulatory patients with milder disease activity, to ensure the model's generalizability across clinical settings. Methodologically, the model should be evolved to process longitudinal data, incorporating temporal features such as cumulative glucocorticoid exposure and fluctuations in DAS28 scores over time. By feeding these dynamic trajectories into the CNN component, future iterations could move from static risk assessment to predicting the precise temporal window of disease progression, ultimately enabling truly preemptive clinical decision-making.

## Supplementary Information

Supplementary Material 1.

## Data Availability

The data underlying this article will be shared on reasonable request to the corresponding author.
