# Supplementary material for: Elucidating osteoporosis response signatures in rheumatoid arthritis using explainable machine learning ensembles
Source: BMC Musculoskelet Disord. 2026 Jan 21;27:148. doi: 10.1186/s12891-026-09526-1 (PMC12905921; doi:10.1186/s12891-026-09526-1)
Supplement: Supplementary file 1 — Supplementary Material 1. [file 12891_2026_9526_MOESM1_ESM.docx]

**Supplementary Table S1. Performance comparison of all models across three classification tasks (AUC).**

| **Model** | **Task 0-1 (Normal vs. Osteopenia)** | **Task 0-2 (Normal vs. Osteoporosis)** | **Task 1-2 (Osteopenia vs. Osteoporosis)** |
| --- | --- | --- | --- |
| **CNN-SVM** | **0.833** | 0.890 | 0.743 |
| XGBoost | 0.748 | 0.910 | 0.729 |
| LightGBM | 0.682 | 0.910 | 0.756 |
| CatBoost | 0.763 | 0.885 | **0.779** |
| Gradient Boosting | 0.537 | 0.890 | 0.681 |
| Random Forest | 0.687 | 0.908 | 0.771 |
| SVC | 0.704 | 0.930 | 0.761 |
| Logistic Regression | 0.685 | **0.935** | 0.723 |
| Naive Bayes | 0.704 | 0.930 | 0.684 |
| KNN | 0.578 | 0.858 | 0.715 |
| Decision Tree | 0.583 | 0.630 | 0.573 |
